# Efficacy and Safety of Adalimumab Biosimilar GP2017 in Patients with Inflammatory Bowel Disease

**DOI:** 10.3390/jcm12216839

**Published:** 2023-10-29

**Authors:** Marta Vernero, Cristina Bezzio, Davide G. Ribaldone, Stefania Costa, Davide Scalvini, Elisa Tribocco, Gianpiero Manes, Simone Saibeni

**Affiliations:** 1 Department of Medical Sciences, University of Turin, 10100 Turin, Italy; marta.vernero@unito.it (M.V.); elisa.tribocco@unito.it (E.T.); 2 IBD Centre, Gastroenterology Unit, Rho Hospital, ASST Rhodense, 20017 Rho, Italy; cristina.bezzio@hunimed.eu (C.B.); davide.scalvini01@universitadipavia.it (D.S.); gmanes@asst-rhodense.it (G.M.); 3 IBD Centre, IRCCS Humanitas, Research Hospital, 20089 Rozzano, Italy; 4 Department of Biomedical Sciences, Humanitas University, 20072 Pieve Emanuele, Italy; 5 Gastroenterology and Digestive Endoscopy Unit, Legnano Hospital, ASST Ovest Milanese, 20025 Legnano, Italy; stefania.costa@asst-ovestmi.it

**Keywords:** adalimumab, biosimilar, GP2017, inflammatory bowel disease, ulcerative colitis, Crohn’s disease

## Abstract

(1) Background: GP2017 is one of the biosimilar drugs of adalimumab, one of the anti-TNF agents used for inflammatory bowel disease (IBD). To date, there is little real-world data about the use of GP2017 in IBD patients. The aim of our study was to evaluate the effectiveness and safety of this biosimilar in an IBD population. (2) Methods: This is an observational retrospective study including patients that were all treated with GP2017 as a first step or as a switch from the originator or other biosimilars. The clinical activity was evaluated at baseline and after 6 and 12 months of therapy. The therapy discontinuation and side effects were also evaluated. (3) Results: a total of 72 patients were included (65 with Crohn’s disease and 7 with ulcerative colitis). Of the 29 patients starting GP2017 as a first adalimumab therapy, clinical remission was achieved in 58.6%. Of the patients starting GP2017 as a switch from the originator (33 patients) or other biosimilars (10 patients), clinical remission was maintained in 78.8% and in 70%, respectively. Regarding the safety, only 11 patients experienced non-serious side effects. During the follow-up, nine patients suspended treatment mainly due to side effects or secondary failure. (4) Conclusions: GP2017 is an effective and safe therapy for IBD patients.

## 1. Introduction

Ulcerative colitis (UC) and Crohn’s disease (CD) are the principal forms of inflammatory bowel disease (IBD). Both represent the chronic inflammation of the gastrointestinal tract, and may cause significant morbidity and an impact on quality of life [1]. The advent of biological therapy has provided relevant improvement in the clinical symptoms, endoscopic lesions, and quality of life in IBD patients [2]. The first class of biologics used in IBD was anti-TNF alpha agents. First of all, infliximab was approved, followed by etanercept, and then adalimumab [3]. It is a fully human immunoglobulin G1 monoclonal antibody (mAb) with subcutaneous administration that inhibits the activity of tumour necrosis factor (TNF)-α [4]. It inhibits the binding between TNF alpha (both the cytoplasmatic and membrane part) and the p55 and p75 TNF receptors, interfering with the cytokine-mediated inflammatory process [5]. Adalimumab has been approved for treating rheumatoid arthritis, ankylosing spondylitis, Crohn’s disease, ulcerative colitis, hidradenitis suppurativa, juvenile idiopathic arthritis, plaque psoriasis, psoriatic arthritis, and uveitis [3]. Although, off-label adalimumab is also administrated for several other rare inflammatory diseases including pyoderma gangrenosum, Behcet disease, Wegener granulomatosis, sarcoidosis, pemphigus, multicentric reticulohistiocytosis, and alopecia areata [3].

As said, one of the major indications of adalimumab is IBD. For instance, the CHARM trial was one of the first clinical trials demonstrating the higher remission rate of CD patients undergoing adalimumab treatment vs. patients receiving a placebo [6]. In the next few years, clinical trials investigating the efficacy of adalimumab in IBD were numerous [7,8,9,10], and their results were then confirmed by meta-analysis [11].

To date, adalimumab is also one of the few biological therapies approved for treating pediatric IBD patients, following the results of clinical trials on children proving the efficacy and safety profile of this drug on this subgroup of patients [12].

Moreover, even though they are both anti-TNF drugs, adalimumab proved efficacy not only in biological naïve patients, but also in patients who previously failed with infliximab or were intolerant to it (as adalimumab is fully humanized, the risk of allergic reactions must be minimized) [13,14].

In addition, given the already cited adalimumab indications in several immune-mediated diseases, adalimumab is the first choice in those patients with extraintestinal manifestations of IBD, especially those with axial spondylarthritis, psoriasis, or uveitis [15].

Adalimumab has as well been reported to effectively prevent post-operative recurrence in Crohn’s disease, showing better results than azathioprine [16,17].

Last but not least, adalimumab is one of the few biological therapies with known efficacy in perianal Crohn’s disease, as it may induce fistula closure [18].

On this note, adalimumab was introduced in both Italian and European guidelines soon after its approval for treating IBD and is still included even in the latest guidelines [19,20,21,22].

In recent years, biosimilars have been developed and increasingly used. Due to their complex structure, biosimilars are biological medicinal products that are similar, but not identical, to an approved biological drug, called the “originator” or “reference product”. According to the European Medicines Agency (EMA), a biosimilar mAb is highly similar to the originator in terms of efficacy, safety, and immunogenicity [23]. In other words, in the European Union (EU), a biosimilar medicinal product is a biological therapy, highly similar to another that is already approved (the “reference drug”), the patent rights of which have expired, and which complies with the principles established by the European Medicines Agency (EMA). Biosimilars are approved according to the same standards of pharmaceutical quality, safety, and efficacy that apply to all biological medicines, for each indication of the reference drug [24]. Thus, with biosimilars, we have in our therapeutical armamentarium new drugs with almost the same efficacy, immunogenicity, and side effects, and with significantly lower costs, which is a great advantage as it gives access to advanced therapies to more and more patients.

Adalimumab GP2017 is the fourth adalimumab biosimilar approved in Europe with the same indications of the originator [25]. The clinical efficacy and safety of multiple switches have been proved through the ADACCESS trial in patients with psoriasis [26]. However, the extrapolation of data across indications is still a matter of debate in the IBD community [4].

Even though some authors demonstrated the safety and efficacy of switching through adalimumab biosimilars [27], data about interchangeability and switching from originators to biosimilars and between biosimilars are still lacking, especially regarding adalimumab [28].

Therefore, the aim of this study was to evaluate the effectiveness and safety of GP2017 in patients with IBD naïve to adalimumab or who switched from the adalimumab originator or from another biosimilar.

Particularly, we aimed to demonstrate our hypothesis that GP2017 has the same efficacy and safety of the adalimumab reference drug, both in patients naïve to adalimumab and in patients who switched from the originator or other biosimilars of adalimumab. Moreover, we hypothesized that patient switching from another form of adalimumab would maintain remission through to follow up. Finally, we did not expect more major side effects than those reported for the reference drug.

## 2. Materials and Methods

This observational, multicentric, retrospective study was conducted in two IBD centers in Northern Italy: Rho Hospital, ASST Rhodense; and Turin Hospital, Città della Salute e della Scienza, Italy. The study protocol was approved by the ethics committee.

Patients were included in the study if they had an established diagnosis of CD or UC, according to ECCO guidelines [29], ongoing or previous therapy with GP2017, and with at least 6 months of follow up available after starting of GP2017.

All medical records were assessed in both centers and all patients meeting inclusion criteria were included in the study.

We enrolled patients in whom GP2017 was the first adalimumab used and patients who received GP2017 as second or third adalimumab. In these patients, time and reason for the switch were also recorded.

Each patient received GP2017 at standard doses. For patients naïve to adalimumab, an induction phase was performed with 160 mg administered subcutaneously and then 80 mg administered subcutaneously after 2 weeks; maintenance phase was performed by means of 40 mg administered subcutaneously every two weeks. For patients switching form originator or from other biosimilar, GP2017 was administered at the standard maintenance dose of 40 mg subcutaneously every 2 weeks.

Dose optimization was defined as the need for weekly subcutaneous administration of GP2017.

For each patient, the following data were collected at baseline: age, sex, smoking habits, previous appendectomy, age at diagnosis, diagnosis of CD or of UC, family history of IBD, presence of extraintestinal manifestations, and previous and current therapy. UC extension and CD location and behavior were assessed according to the Montreal criteria [30,31].

We considered three time points: start of therapy (T0), 6 months (T1), and 12 months (T2). At each time point, disease activity was evaluated through Harvey–Bradshaw index (HBI) [32] for CD and through partial Mayo (pMayo) [33] for UC.

Primary endpoints of the study were the following:

- Achievement of clinical remission at 12 months in IBD patients starting GP2017 as first adalimumab;
- Maintenance of clinical remission at 12 months in IBD patients starting GP2017 as switch therapy from other adalimumab.
- Secondary endpoints were the following:
- Achievement of clinical remission at 6 months in IBD patients starting GP2017 as first adalimumab;
- Maintenance of clinical remission at 6 months in IBD patients starting GP2017 as switch therapy from other adalimumab;
- Achievement of steroid-free clinical remission at 6 and 12 months in IBD patients starting GP2017 as first adalimumab;
- Maintenance of steroid-free clinical at 6 and 12 months in IBD patients starting GP2017 as switch therapy from other adalimumab;
- Therapy persistence;
- Rate of adverse events;
- Influence of perianal disease, smoking, presence of other immune-mediated inflammatory diseases (IMID), or previous anti TNF therapies (infliximab or other adalimumab) on reaching or maintaining remission. This analysis was performed on the whole population;
- Influence of side effects and remission at 6 and 12 months on therapy persistence (this analysis was performed on the whole population);
- Need for optimization and influence of previous anti TNF exposure, smoking, and presence of extraintestinal manifestations on need for optimization (this analysis was performed on the whole population).

Clinical remission was defined as HBI < 5 for CD or pMayo < 2 for UC. Steroid-free remission was defined as remission without ongoing steroid therapy. Following the non-response imputation paradigm, patients who interrupted therapy before the end of follow-up were considered as failure.

### Statistical Analysis

Continuous variables were tested for normal distribution through D’Agostino Pearson’s test. Mean and standard deviation (SD) were used for normally distributed variables, while median and inter-quartile range 25–75% (IQR) were used for non-normally distributed variables. Differences between two groups were tested through Student’s *t*-test for variables with normal distribution and through Wilcoxon’s test for variable with non-normal distribution. For categorical variables, frequencies were expressed in percentages. Differences between groups were tested by means of McNemar’s test. Correlation was tested with logistic regression at univariate analysis and through multiple regression for significant variables at multivariate analysis.

All data were collected in an excel database and analysed with Med Calc Software version 16 (MedCalc Software Ltd., Ostend, Belgium). Statistical significance was set for *p* values < 0.05, and confidence intervals were calculated at 95%.

## 3. Results

Seventy-two patients were included in the study. The overall baseline characteristics are summarized in Table 1.

Twenty-nine patients (40.3%) started GP2017 as their first adalimumab therapy, 33 patients (45.8%) were first treated with the adalimumab originator, and 10 (13.8%) with another adalimumab biosimilar (ABP 501 and SB5).

### 3.1. Efficacy of GP2017 as a First Therapy

Twenty-nine patients started GP2017 as their first adalimumab. GP2017 was started for steroid-dependence in 16 patients (55.2%), steroid-resistance in 5 (17.2%), failure/intolerance to azathioprine in 2 (6.9%), for preventing or treating post-operative recurrence in 4 patients (13.8%), for perianal disease in 1 patient (3.4%), and for associated rheumatologic or dermatological indications in 1 patient (3.4%).

**Figure 1:**
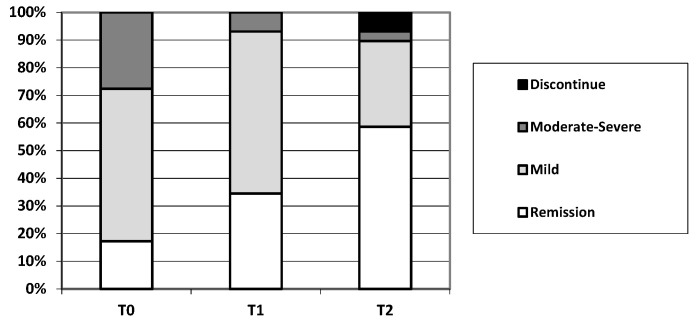
Change in clinical disease activity following starting of GP2017 therapy in IBD patients.

Regarding the GP2017 efficacy, at the time of starting the therapy (T0), 5/29 patients (17.2%) were in clinical remission, 16/29 patients (55.1%) had mild disease activity, and 8/29 patients (27.6%) had moderate-to-severe activity. After 6 months (T1), 10/29 (34.5%) were in remission, 17/29 (58.6%) had mild activity and 8/29 patients (27.6%) had moderate-to-severe activity. After 12 months (T2), 17/29 (58.6%) were in remission, 9/29 (31.0%) had mild activity, and 1/29 patients (3.5%) had moderate-to-severe activity (Figure 1).

Thus, at T0, 5/29 (17.2%) of patients were in remission, while at T6, 10/29 (34.5%) patients reached remission (*p* = 0.0005), and at T12, 17/29 (58.6%) were in remission (*p* = 0.001).

### 3.2. Efficacy of GP2017 after Adalimumab Originator

In our population, 33 patients switched from the adalimumab originator to GP2017 (T0). In this group, when GP2017 was started, 25/33 patients (75.8%) were in clinical remission, 6/33 patients (18.2%) had mild activity, and 2/33 (6.0%) had moderate-to-severe activity. The median time from starting adalimumab and the switch to GP2017 was 12.5 months (IQR 0.00–38.0).

At 6 months after the switch (T1) to GP2017, 26/33 patients (78.8%) were in clinical remission, 4/33 patients (12.1%) had mild activity, and 1/33 (3.0%) had moderate-to-severe activity.

At 12 months after the switch (T2), 26/33 patients (78.8%) were in clinical remission, 2/33 patients (6.1%) had mild activity, and 0/33 (0.0%) had moderate-to-severe activity.

**Figure 2:**
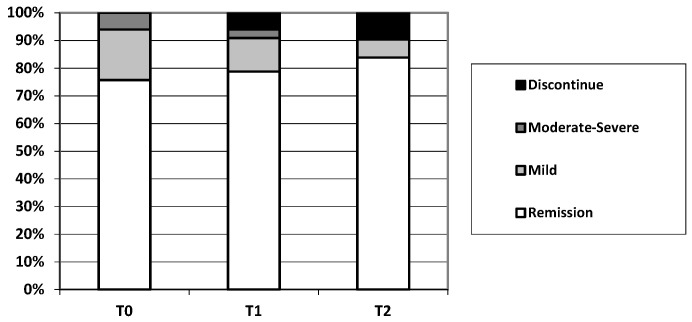
Change in clinical disease activity following a switch from the originator to GP2017 in IBD patients.

The change in the clinical disease activity is represented in Figure 2.

Thus, at T0, 25/33 patients (75.8%) were in clinical remission, while after 6 months of GP2017, 26/33 (78.8%) (*p* = 0.5) were in remission, and the same percentage was maintained after 12 months (*p* = 0.5).

### 3.3. Efficacy of GP2017 after Other Adalimumab Biosimilar

In patients with a switch from other adalimumab biosimilars (T0), when GP2017 was started, 6/10 patients were in clinical remission, 3/10 patients had mild activity and 1/10 had moderate-to-severe activity. The median time from starting adalimumab and switching to GP2017 was 11.5 months (IQR 0.00–34.0).

At 6 months after the switch to GP2017 (T1), 7/10 patients were in clinical remission, 2/10 patients had mild activity and 1/10 had moderate-to-severe activity.

At 12 months after the switch (T2), 7/10 patients were in clinical remission, 2/10 patients had mild activity and 0/10 had moderate-to-severe activity.

**Figure 3:**
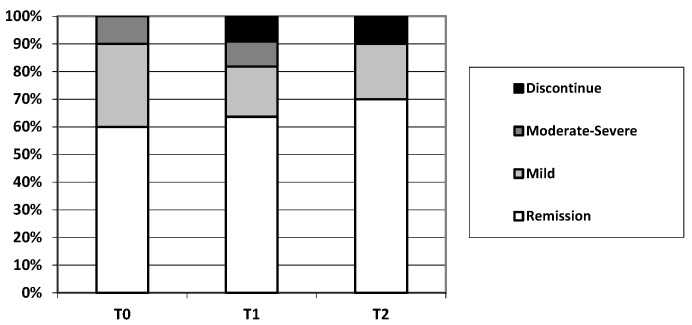
Change in clinical disease activity following a switch from the other adalimumab biosimilar to GP2017 in IBD patients.

The change in the clinical disease activity is represented in Figure 3.

Thus, at T0, 6/10 (60%) patients were in remission at the time of switching, 7/10 patients after 6 months (*p* = 1), and the same proportion after 12 months (*p* = 1).

### 3.4. Steroid-Free Remission

**Figure 4:**
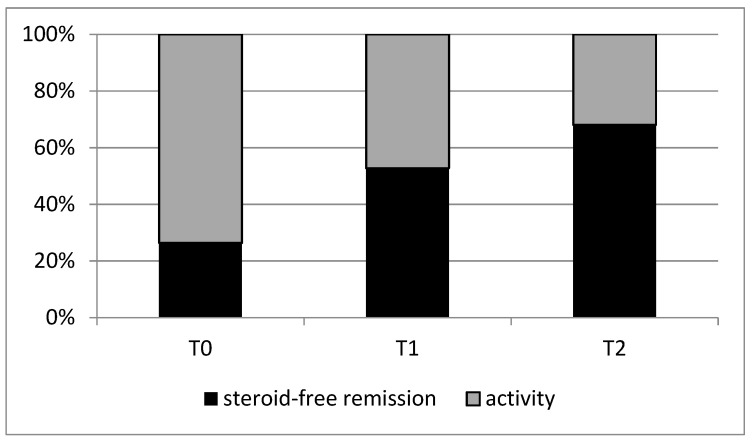
Steroid-free remission at T0, T1 and T2.

As shown in Figure 4, the overall steroid-free remission was present in 19 patients at T0 (26.4%), in 38 patients (52.8) at T1 (*p* < 0.001), and in 49 (68.1%) at T2 (*p* < 0.001).

### 3.5. Factors Influencing Remission

For the univariate analysis, the whole population was considered altogether.

As far as the coexistence of at least one other extraintestinal manifestation (EIM) is concerned, the odds ratio (OR) for reaching remission at 12 months when one or more EIMs were present was 0.79 (95% CI 0.20–3.01; *p* = 0.80). Regarding smoking habits, the OR for reaching remission at 12 months for the active smokers compared to the non-smokers or former smokers was 1.63 (95% CI 0.44–6.01; *p* = 0.90). Moreover, the patients who switched from another adalimumab formulation tended to have a higher probability of reaching remission, with an OR of 2.25 (95% CI 0.61–8.20; *p* = 0.69). On the other hand, the presence of perianal disease seemed to decrease the probability of remission at 12 months, with an OR of 0.44 (95% CI 0.11–1.87; *p* = 0.10). As regards previous therapy, none of the analyzed (infliximab, vedolizumab, or ustekinumab) demonstrated an influence on the clinical remission at 12 months; in particular, previous treatment with infliximab seemed to influence remission at 12 months (OR 1.20; 95% IC 0.37–3.20; *p* = 0.51).

### 3.6. Safety of GP2017 Therapy

Regarding the safety, 11 (15.2%) patients experienced side effects; particularly, seven in the first 6 months of therapy and four in the following 12 months. However, none of these occurrences were severe; Table 2 summarizes the observed side effects.

### 3.7. Therapy Persistence

**Figure 5:**
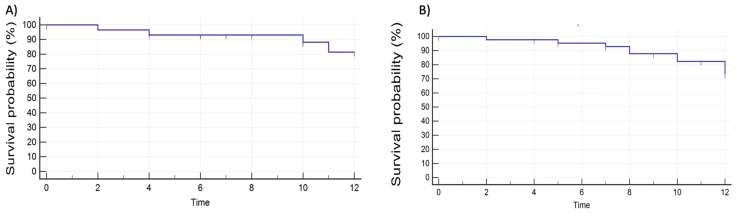
Kaplan–Meier curve of therapy persistence. (**A**) Therapy persistence in patients naïve to adalimumab. (**B**) Therapy persistence in patients switching from another adalimumab.

In the first 6 months, 3/72 patients (4.2%) discontinued therapy: two due to secondary failure, and one due to side effects. During the following 6 months, 6/69 patients (8.7%) discontinued therapy: three for secondary failure, two due to side effects, and one due to pregnancy. Figure 5 shows the Kaplan–Meier curve of the therapy persistence in the patients starting with GP2017 (Figure 5A) and in patients switching from another adalimumab (Figure 5B). The percentages of therapy persistence are similar in patients starting GP2017 as their first therapy and in patients starting GP2017 as a switch from another adalimumab.

### 3.8. Factors Influencing Therapy Persistence

We did not find any factor significantly influencing treatment persistence. Only the patients who experienced side effects tended to be more likely to suspend GP2017 (OR 3.6; 95% CI 0.98–13.8; *p* = 0.06).

### 3.9. Need for Optimization

Overall, in our population, nine patients out of 72 (12.5%) needed dose optimization of GP2017; among these, seven in the first 6 months of therapy, and two between 6 and 12 months of therapy. Among the 43 patients who switched from another adalimumab (33 from the originator, 10 from other biosimilars), optimization was needed in no one at 6 months and in three (6.9%) at 12 months.

As regards influencing factors, previous infliximab exposure significantly increased the risk of optimization: OR 4.4 (95% CI 1.27–17.3, *p* = 0.037). Similarly, the absence of a previous adalimumab therapy was protective towards the need for optimization, with an OR of 0.24 (95% CI 0.06–1.02, *p* = 0.04). On the other hand, smoking habits did not show a significant influence on the optimization rate (OR 0.20; 95% CI 0.10–2.7; *p* = 0.70), and neither did the presence of at least one extraintestinal manifestation (OR 1.15; 95% CI 0.27–4.82; *p* = 0.84). Finally, the presence of perianal disease did not influence the risk of optimization (OR 0.65; 95% CI 0.13–3.34; *p* = 0.59).

Previous infliximab exposure and the absence of a switch from another adalimumab were included in the multivariate analysis; only the influence of previous infliximab exposure was confirmed as significantly associated with the need for optimization (OR 4.2; 95% CI 1.02–17.34; *p* = 0.04)

## 4. Discussion

The advent of biosimilars marked a turning point in the treatment of IBD, as they significantly reduced the costs of advanced therapies and allowed their wide spreading. Several real-life studies on the use of adalimumab biosimilars in IBD have been recently published; however, data about GP2017 are still scarce.

In our series, as expected, the majority of patients started GP2017 adalimumab for steroid-dependence or steroid-resistance, or for post-operative recurrence treatment or prevention, which are the major indications for biological therapy in IBD [22]. Regarding the primary outcome, in the IBD patients starting GP2017 as their first adalimumab, the remission rate at 12 months was achieved in around 60% of the patients. This finding is coherent with previous data on the efficacy both for the adalimumab originator and other adalimumab biosimilars [34,35,36,37]. Among the patients who started GP2017 switching from the adalimumab originator or biosimilars, the primary outcome, which is maintenance of remission, was observed in around 80% of the patients. Again, these findings are in line with data reported from other authors [34,35,36,37]. Of note, previous adalimumab therapy did not influence the response at 12 months, confirming that switching from the originator or from other adalimumab biosimilars is safe and effective [4,27,28].

Moreover, the data on the clinical remission at 6 months and the rates of steroid-free remission are not different from what is already known [34,35,36,37].

As for the safety profile, only 11 patients experienced non-serious side effects including headache, allergic reactions, arthralgia, and cutaneous manifestations. This is coherent with the known major side effects of adalimumab [38,39].

The retention rate of GP2017 was high in our population; indeed, during follow-up, nine patients suspended treatment mainly due to side effects or secondary failure, in line with what is currently reported in the literature [40,41,42,43]. The concept of therapy persistence is an emerging issue in the management of IBD. Since this is a chronic, non-curable condition, with a high economic and social burden, continued therapy is crucial for the long-term achievement of the therapeutic aims. Moreover, it has been demonstrated that overall costs are higher in nonpersistent patients compared to the persistent population [44]. Interestingly, despite smoking being an ascertained risk factor for CD and a worse clinical course of the disease [45], it did not seem to influence the GP2017 persistence in therapy or the need of optimization, suggesting that the response to this drug is not influenced by smoking.

In our population, the optimization rate was around 12.5%, in line with previous observations [46]. At the multivariate analysis, only previous exposure to infliximab resulted in being significantly associated with the need for optimization. This finding is not surprising, since it is known that patients previously treated with an anti-TNF agent (often withdrawn because of failure) are expected to be less likely to respond to standard doses [47,48].

The present study has several limitations. First of all, its observational nature may have influenced the data collection, with some data missing as they were not reported in the medical charts. One other important limitation is the lack of a control group, which does not allow us to draw generalizable conclusions (however, we chose not to have a control group as it was difficult to identify a suitable retrospective population for this purpose). Of note, the sample size was not so high and a relatively small number of patients was affected by UC. This could be due to the fact that, among the anti-TNF class of drugs, infliximab is often preferred in ulcerative colitis patients as it has shown a better efficacy [49]; subsequently, the majority of patients undergoing adalimumab are affected by Crohn’s disease. In addition to that, a relatively small number of patients were switched from other adalimumab biosimilars, so this subgroup analysis was led by a small sample size, which may have affected the statistics. This may be mainly because, when possible, we tend not to switch from a biosimilar to another in order to avoid multiple switches. Moreover, laboratory, radiological, and endoscopic data are lacking in our cohort, as, due to the retrospective and observational nature of the study, we did not have homogeneous laboratory test timepoints and instrumental evaluation was different in the time and technique for each patient, so they were incomparable.

Our study confirms the efficacy of the adalimumab biosimilar GP2017 in the management of IBD patients. Moreover, switching from adalimumab biosimilar or originator did not influence the outcome, confirming what we already know about the efficacy and safety of other adalimumab biosimilars in IBD [27]. Also, we provide further evidence about the therapeutic effectiveness of the adalimumab biosimilar GP2017. Its reduced costs provide the opportunity to receive benefit of these advanced therapies to a growing number of patients worldwide.

## 5. Conclusions

Adalimumab GP2017 is an effective treatment in IBD in a real-world setting. Moreover, the retention rate and safety profile are comparable to those present in the literature regarding the originator, justifying the feasibility to switch from the originator to a biosimilar or from biosimilar to biosimilar.

## Figures and Tables

**Table 1:** Features at starting therapy with GP2017.

AGE in years (mean (SD))	45.1 (13.4)
AGE AT DIAGNOSIS in years (mean (SD))	33.6 (12.4)
CROHN’S DISEASE N (%)	65 (90.3%)
DISEASE DURATION in years (median (IQR))	9 (5–17)
SEX (M) N (%)	39 (54.2%)
SMOKE N (%)	Never 40 (55.5%) Former 2 (2.8%) Active 30 (41.7%)
PERIANAL DISEASE N (%)	17 (23.6%)
FAMILY HISTORY OF IBD N (%)	9 (12.5%)
PPREVIOUS THERAPY N (%)	Immunosuppressants 21 (29.2%) Infliximab 14 (19.4%) Ustekinumab or Vedolizumab 5 (6.9%)
UC EXTENSION N (%)	E2 4 (57.1%) E3 3 (42.9)
CD LOCATION N (%)	L1 25 (38.5%) L2 6 (9.2%) L3 32 (49.2%) L4 4 (6.1%)
CD BEHAVIOUR N (%)	B1 27 (41.5%) B2 16 (24.6%) B3 22 (33.9%)
EXTRA-INTESTINAL MANIFESTATIONS N (%) (more than one for patient)	None 50 (69.4%) Cutaneous 4 (5.5%) Peripheral arthritis 9 (12.5%) Axial arthritis 10 (13.9%) Ocular 2 (2.8%)

SD = standard deviation; IQR = inter-quartile range; CD = Crohn’s disease; IBD = inflammatory bowel disease; UC = ulcerative colitis.

**Table 2:** GP2017 side effects.

SIDE EFFECT	N (%)
Allergy	3 (4.2%)
Cutaneous	4 (5.5%)
Articular	1 (1.4%)
Headache	1 (1.4%)
Other	2 (2.8%)

## Data Availability

Data are available upon reasonable request.
